# Pretreatment serum markers and lymphocyte response to interleukin-2 therapy

**DOI:** 10.1038/sj.bjc.6690371

**Published:** 1999-05-01

**Authors:** L Fumagalli, P Lissoni, G Di Felice, S Meregalli, G Valsuani, S Mengo, F Rovelli

**Affiliations:** Chiron Italia, Milano, Italy; Division of Radiation Oncology, andLaboratory, S Gerardo Hospital, 20052 Monza (Milano), Italy

**Keywords:** IL-2, immunotherapy, IL-6, neopterin, sIL-2R, lymphocytosis

## Abstract

Lymphocytosis is a marker of subcutaneous interleukin (IL)-2 therapy efficacy, whereas baseline elevated inflammatory indices were noticed in IL-2-resistant disease. The aim of this study was to analyse the relationship between pretreatment circulating values of IL-6, neopterin, sIL-2R, ESR and the changes in lymphocyte number in response to IL-2 administration. Twenty metastatic renal cell cancer patients were treated with subcutaneous IL-2 immunotherapy (6 000 000 IU day^−1^ for 6 days per week for 4 weeks); tumour response consisted of partial response (PR) in four patients, stable disease (SD) in eight patients and progressive disease (PD) in eight patients. Abnormally high pretreatment values of each marker were found as follows: IL-6 in seven patients, neopterin in nine patients, sIL-2R in 13 patients. In response to IL-2 immunotherapy, a significantly higher mean increase in lymphocyte number and a higher percentage of patients with tumour response or stable disease were observed when pretreatment values of IL-6, neopterin and sIL-2R were within the normal range, in comparison to patients with high values for these markers. The pretreatment excess of these serum inflammatory markers seems to negatively influence both the host and tumour response to IL-2 administration, by preventing the IL-2-induced lymphocytosis and resulting in tumour progression. Further studies are requested to verify if overall survival and quality of life may depend on pretreatment host immune status and/or lymphocyte response after IL-2 administration. © 1999 Cancer Research Campaign


*British Journal of Cancer* (1999) **80**(3/4), 407–411
© 1999 Cancer Research Campaign
Article no. bjoc.1998.0371

### Pretreatment serum markers and lymphocyte response to interleukin-2 therapy

**L Fumagalli[1], P Lissoni[2], G Di Felice[1], S Meregalli[2], G Valsuani[1], S Mengo[2] and F Rovelli[3]**

[1]Chiron Italia, Milano, Italy; [2]Division of Radiation Oncology and [3]Laboratory, S Gerardo Hospital, 20052 Monza, (Milano), Italy

**Summary** Lymphocytosis is a marker of subcutaneous interleukin (IL)-2 therapy efficacy, whereas baseline elevated inflammatory indices were noticed in IL-2-resistant disease. The aim of this study was to analyse the relationship between pretreatment circulating values of IL-6, neopterin, sIL-2R, ESR and the changes in lymphocyte number in response to IL-2 administration. Twenty metastatic renal cell cancer patients were treated with subcutaneous IL-2 immunotherapy (6 000 000 IU day$^{-1}$ for 6 days per week for 4 weeks); tumour response consisted of partial response (PR) in four patients, stable disease (SD) in eight patients and progressive disease (PD) in eight patients. Abnormally high pretreatment values of each marker were found as follows: IL-6 in seven patients, neopterin in nine patients, sIL-2R in 13 patients. In response to IL-2 immunotherapy, a significantly higher mean increase in lymphocyte number and a higher percentage of patients with tumour response or stable disease were observed when pretreatment values of IL-6, neopterin and sIL-2R were within the normal range, in comparison to patients with high values for these markers. The pretreatment excess of these serum inflammatory markers seems to negatively influence both the host and tumour response to IL-2 administration, by preventing the IL-2-induced lymphocytosis and resulting in tumour progression. Further studies are requested to verify if overall survival and quality of life may depend on pretreatment host immune status and/or lymphocyte response after IL-2 administration.

**Keywords:** IL-2; immunotherapy; IL-6; neopterin; sIL-2R; lymphocytosis

After more than 10 years of clinical studies on interleukin (IL)-2 anti-tumour activity, it has been shown that both the activity and the efficacy of IL-2 cancer immunotherapy on tumour response and on overall survival, respectively, do not depend upon tumour characteristics alone, but also on the host's general status (Palmer et al, 1993) and immune status (Blay et al, 1992) prior to IL-2 therapy. With regard to pretreatment immune status, some large studies (Blay et al, 1992; Lopez-Hanninen et al, 1996) have reported a correlation between high inflammatory activation prior to the onset of treatment, such as abnormally high pretreatment values of IL-6, C reactive protein (CRP), or elevated erythrosedimentation rate (ESR), and tumour resistance to IL-2 activity.

Neopterin, a specific marker of macrophage activation, (Diamondstone et al, 1994) and circulating levels of soluble IL-2 receptor (sIL-2R), whose main source would be T-cells (Rubin et al, 1985), could be two other important parameters of the inflammatory response activation and may, in fact, suggest the involvement of both macrophages and lymphocytes in the physiopathology of cancer-induced immune unbalance.

So far, most studies have evaluated the prognostic significance of a single immune index on IL-2 immunotherapy (Jeal and Goa, 1997). Therefore, the physiopathology of mechanisms responsible for the prognostic significance of baseline levels of a single cytokine, or of other immune markers after exogenous IL-2 administration, have still to be thoroughly investigated and established. Among possible mechanisms, the documented inhibitory effect of IL-6 on IL-2-induced generation of cytotoxic lymphocytes (Kishimoto, 1989) may be responsible for tumour resistance to IL-2 therapy in patients with high levels of endogenous IL-6. In contrast, the lack of IL-2 anti-tumour activity in the presence of pretreatment elevated blood levels of neopterin (Lissoni et al, 1994) and/or sIL-2R (Gooding et al, 1995) is more difficult to explain, as the increased levels of neopterin and sIL-2R reflect the activation of the immune system in either an anti-tumour or an immunosuppressive way (Rubin et al, 1985; Diamondstone et al, 1994; Jeal and Goa, 1997). At present, it is still unclear whether the association among high pretreatment circulating IL-6, neopterin and sIL-2R, and resistance to IL-2 anti-tumour activity may reflect different unrelated cytokine alterations or, on the contrary, whether the association may outline a common pathway responsible for the deficiency of the anti-tumour immune activation.

With regard to host response after IL-2 administration, it is known that lymphocytes play a fundamental role in mediating tumour cell destruction (Grimm et al, 1982) and that rebound lymphocytosis represents the typical biomarker of immune activation in response to IL-2 injection (Palmer et al, 1993). Furthermore, in patients with metastatic renal cell cancer who achieve an objective response, a higher lymphocyte increase has been observed after IL-2 subcutaneous (s.c.) therapy (Gohring et al, 1996). On this basis, the present study was carried out to evaluate host response after IL-2 s.c. low-dose immunotherapy in metastatic cancer patients, measured as post-treatment changes in lymphocyte number, in relation to the pretreatment values of IL-6, neopterin, sIL-2R and ESR.

*Received 19 May 1998*
*Revised 27 October 1998*
*Accepted 19 November 1998*

*Correspondence to:* P Lissoni

### PATIENTS AND METHODS

From January to October 1996, 20 consecutive metastatic renal cell cancer (RCC) patients entered the study. The characteristics of

**Table 1** Clinical characteristics of metastatic renal cell cancer patients

| | |
|---|---|
| *n* | 20 |
| M/F | 12/8 |
| Median age (years) | 59 (45–76) |
| Median PS (Karnofsky %) | 90 (80–100) |
| Dominant metastatic sites | |
| Lung | 12 |
| Bone | 5 |
| Liver | 3 |

**Table 2** Mean increase in total lymphocyte number in relation to the pretreatment values of IL-6, neopterin, sIL-2R and ESR

| Pretreatment marker value | Lymphocyte increase during IL-2 | | |
|---|---|---|---|
| | no. of patients | (*n*-mmc) | *P* |
| IL-6 | | | |
| High | 7/20 | 678 ± 82 | |
| Normal | 13/20 | 2543 ± 121 | < 0.001 |
| Neopterin | | | |
| High | 9/20 | 1132 ± 237 | |
| Normal | 11/20 | 2369 ± 214 | < 0.025 |
| sIL-2R | | | |
| High | 13/20 | 1332 ± 169 | |
| Normal | 7/20 | 2458 ± 342 | < 0.05 |
| ESR | | | |
| > 40 mm $h^{-1}$ | 14/20 | 1456 ± 353 | |
| < 40 mm $h^{-1}$ | 6/20 | 2013 ± 195 | NS |

NS, not significant.

**Table 3** Pretreatment IL-6 serum values in relation to neopterin and sIL-2R values

| Baseline marker value | IL-6 value (pg $ml^{-1}$) | *P* |
|---|---|---|
| Neopterin | | |
| High | 94 ± 19 | |
| Normal | 34 ± 8 | < 0.05 |
| sIL-2R | | |
| High | 89 ± 21 | |
| Normal | 17 ± 3 | < 0.025 |

patients are reported in Table 1. Eligibility criteria were: hystologically proven metastatic renal cell cancer, measurable disease, first-line therapy of the metastatic stage, previous nephrectomy, age less than 80 years, a performance status (PS) according to Karnofsky score greater than 70%, no concomitant chronic therapy with steroids or other drugs influencing the immune system, no important illnesses other than cancer, no brain metastasis and no second tumour.

Recombinant human IL-2 was injected s.c. at a dose of 3 000 000 IU twice daily for 6 days per week for 4 consecutive weeks, corresponding to one complete immunotherapeutic cycle. The clinical response was evaluated according to WHO criteria. In non-progressing patients, a second cycle was administered after a 21-day rest. Following this, patients underwent a maintenance schedule consisting of treatment 6 days per month, until progression or toxicity. Routine laboratory tests, including differential count, were performed before the onset of IL-2 treatment and at 1-week intervals until the end of the cycle. Venous blood samples were collected the morning before the onset of therapy. Serum levels of IL-6 and sIL-2R were measured by an enzyme immunoassay using commercially available kits. Intra-assay and interassay coefficients of variation, established by at least 20 consecutive assays, were less than 5% and 7% for IL-6 and less than 5% and 6% for sIL-2R respectively. Serum levels of neopterin were measured by radioimmunoassay using commercially available kits. Intra-assay and interassay coefficients of variation were less than 4% and 5% respectively. Normal values obtained in our laboratory (95% confidence limits) in 20 healthy subjects were as follows: IL-6 < 31 pg $ml^{-1}$; neopterin < 2.5 ng $ml^{-1}$; sIL-2R < 920 U $ml^{-1}$; ESR < 20 mm per hour; lymphocyte count > 1000 mmc. Results were reported as the mean ± s.e.m. Changes in lymphocyte mean number were expressed as mean maximum increase observed during the first immunotherapeutic cycle. Data were statistically analysed by the $\chi^2$ test, Student's *t*-test and analysis of variance, as appropriate.

### RESULTS

Tumour objective response consisted of partial remission (PR) in 4/20 patients (20%), with a median duration of 8+ months (range: 4–11+ months). Stable disease (SD) was observed in 8/20 (40%) patients, whereas the remaining eight patients showed progressive disease (PD) in response to the first immunotherapeutic cycle.

The following immunoinflammatory serum markers were found to be at abnormally high pretreatment levels respectively: IL-6 in 7/20 (35%) patients, neopterin in 9/20 (45%) patients and sIL-2R in 13/20 (65%) patients. ESR baseline values greater than 40 mm $h^{-1}$ were observed in 14/20 (70%) patients.

Mean increase in lymphocyte number occurring in patients during IL-2 administration, in relation to the pretreatment values of IL-6, neopterin, sIL-2R and ESR are shown in Table 2. The mean increase in lymphocyte number shown by patients having normal pretreatment values of IL-6, neopterin and sIL-2R was significantly higher than that observed in patients having elevated values of the same markers. For ESR, the lymphocyte increase in patients with a baseline value < 40 mm $h^{-1}$ was higher than in patients having a baseline value > 40 mm $h^{-1}$, but not significantly.

The relationship of pretreatment values of IL-6 to neopterin and sIL-2R levels is shown in Table 3. It is noted that IL-6 mean serum values were significantly higher in patients having previously elevated neopterin values vs normal, as well as in patients with high sIL-2R concentrations vs normal.

Regarding objective tumour response, a higher percentage of patients having elevated baseline values of the immunoinflammatory markers (respectively IL-6, neopterin, sIL-2R and ESR) showed tumour progression, whereas patients having baseline values within normal range for the same markers showed a higher rate of non-progressing disease (PR ± SD). Details are shown in Table 4.

### DISCUSSION

Elevated values of a specific inflammatory markers, such as CRP and ESR, are frequent in advanced cancer patients with poor prognostic characteristics, such as cachexia (Scott et al, 1996).

 © Cancer Research Campaign 1999

**Table 4** Tumour objective response in relation to normal or elevated pretreatment values of IL-6, neopterin, sIL-2R and ESR in 20 metastatic renal cell cancer patients

| Marker Pretreatment value | no. of patients | Tumour response (*n*) CR | PR | SD | PD | OR ± SD (%) | *P* |
|---|---|---|---|---|---|---|---|
| IL-6 | | | | | | | |
| High | 7 | – | – | 2 | 5 | 2/7 (29) | |
| Normal | 13 | – | 4 | 6 | 3 | 10/13 (77) | < 0.01 |
| Neopterin | | | | | | | |
| High | 9 | – | 1 | 2 | 6 | 3/9 (33) | |
| Normal | 11 | – | 3 | 6 | 2 | 9/11 (82) | < 0.05 |
| sIL-2R | | | | | | | |
| High | 13 | – | 2 | 4 | 7 | 6/13 (46) | |
| Normal | 7 | – | 2 | 4 | 1 | 6/7 (86) | < 0.05 |
| ESR | | | | | | | |
| > 40 mm $h^{-1}$ | 14 | – | 1 | 6 | 7 | 7/14 (50) | |
| ≤ 40 mm $h^{-1}$ | 6 | – | 3 | 2 | 1 | 5/6 (83) | < 0.05 |

Moreover, it is already known that lymphocytopenia (Stanley, 1980) and/or low lymphocyte percentage (Maltoni et al, 1997) are independent predictive factors for short survival in cancer patients, whereas a higher lymphocyte number is related to longer survival (Riesco, 1970; Kim et al, 1976; Stanley, 1980). Until recently, the underlying mechanisms responsible for the debilitation of the host immune status could not be explained. Recent advances in understanding the biology of the tumour in relation to the host (Yoshino et al, 1992) demonstrate that this immune imbalance can in part be explained by the decreased production of endogenous IL-2 (Monson et al, 1986; Lissoni et al, 1989). Further, enhanced production of a number of agents, including IL-6 itself, IL-10 (Koo et al, 1992; Gastl et al, 1993), prostaglandin E2 (Chouaib and Fradelizi, 1982) and (TGF-β) may exert either a suppressive or pro-inflammatory activity, resulting in inhibition of IL-2-induced anti-tumour activity (Sedlmayr et al, 1991; Fischer et al, 1995).

Previous large scale studies on IL-2 treatment have shown that pretreatment high inflammatory activation, measured by markers such as IL-6 and CRP, predicts lack of IL-2 activity on tumour objective response and its efficacy on overall survival in metastatic cancer patients (Blay et al, 1992; Tartour et al, 1994). However, a separate, smaller study demonstrated that detectable IL-6 serum levels correlated with paraneoplastic syndromes but not with response or survival (Walther et al, 1998). Regarding lymphocyte count changes, some studies (Palmer et al, 1993; Gohring et al, 1996) demonstrated that a greater lymphocyte increase after s.c. IL-2 administration occurrs in metastatic renal cell cancer patients with objective response. According to preliminary data, a greater lymphocyte increase is observed in patients with peak of endogenous IL-12 serum levels ≥ 100% vs baseline after s.c. IL-2 administration (Lissoni et al, 1998).

The results of our study confirm that pretreatment inflammatory activation, measured as circulating abnormal levels of IL-6, neopterin and sIL-2R, negatively influences the host response to IL-2 administration, represented by the lack of rebound lymphocytosis. Moreover, IL-6 values are higher in patients who show previously elevated blood levels of neopterin and/or sIL-2R.

The involvement of dominant metastasis site in influencing the host response could not be assessed in this study, as the number of patients within each dominant metastasis category were too low to make any correlation between metastasis site and serum markers. Indeed, further larger studies will be required to establish whether the increase in lymphocyte number in relation to the clinical response may be influenced by the site of disease.

The findings of this study may be considered with regard to both the biological and the clinical aspects of the immune physiopathology of cancer. Immunobiologically, these observations suggest that the inflammatory activation in metastatic cancer is likely to be multifactorial, involving at least both macrophages and lymphocytes, as indicated by neopterin and sIL-2R respectively. Secondarily, the inflammatory pathway activation may influence the function of the whole lymphatic compartment, as represented by unpaired lymphocyte growth (and/or lymphocyte recirculation) after stimulation with IL-2.

The high levels of circulating sIL-2R and IL-6 would directly inhibit the physiologic IL-2-dependent immune cell response as demonstrated both in vitro and in vivo. The circulating sIL-2R would act by the binding of circulating IL-2 (Rubin et al, 1986) and abrogating the activation of peripheral mononuclear cells (Zorn et al, 1994; Gooding et al, 1995), whereas IL-6 is able to inhibit both the natural killer activity (Tanner and Tosato, 1991) and the lymphokine-activated killer cell activity (Scheid et al, 1994). Finally, IL-6 may act as autocrine tumour growth factor in renal cell carcinoma (Koo et al, 1992).

The negative effect of the macrophage marker neopterin (Diamondstone et al, 1994) on IL-2-dependent immune function might be related to the suppressive activity exerted by macrophages in malignancy (Naor and Duke Cohan, 1986), as we are not aware of direct influence of neopterin on IL-2 activity. Indeed, high pretreatment values of neopterin may reflect a pathologically unfavourable activity of macrophages in cancer, possibly exerted through other mediators; in agreement with this hypothesis, the poor prognostic value of high serum levels of macrophage colony-stimulating factor (M-CSF) in patients with lung cancer was recently described (Katsumata et al, 1996).

Regarding clinical aspects, the present study suggests that elevated pretreatment inflammatory status negatively influences not only the biological effects, but also the therapeutic activity exerted by IL-2 on both the host and the tumour, represented by the impairment of lymphocyte rebound and by tumour progression following IL-2 administration respectively.

© Cancer Research Campaign 1999 

Unlike studies involving chemotherapeutic drugs in which only objective tumour regression is considered because only tumour destruction – mainly complete response – following antiblastic agents administration is generally associated with improvement of survival and quality of life, in our study the tumour response and the stable disease were considered together as therapeutic effects of IL-2 treatment. In fact, unlike chemotherapeutic agents, IL-2 acts to boost the host defense system and provides clinical benefit in overall survival (patient outcome) not only following tumour disappearance, but also in patients achieving stable disease, whose overall and median survival is in fact reported to be equal to those of partial responders (Schoof et al, 1993; Buzio et al, 1997; Figlin et al, 1997).

The possibility to predict the host response to cytokine treatment might play an important role in the clinical planning of treatments. In fact, the characterization of the pretreatment inflammatory status would influence the choice of patients suitable for IL-2 therapy, or would identify the most appropriate time to start IL-2 treatment, e.g. only after effective suppression of the inflammatory pathways.

Indeed, the identification of biomarkers other than the tumour response able to predict the patient survival (American Society of Clinical Oncology, 1996) would allow the design of therapeutic strategies using appropriate cytokine administration. This would verify if the therapeutic rebalance of the host immune system (Wojtowicz-Praga, 1997) may improve patient outcome in cancer. The results of previous, and the present, studies suggest that both pretreatment inflammatory markers – such as IL-6, neopterin and sIL-2R – and the host response after IL-2 administration (i.e. lymphocytosis) are worth further studies as to their value as potential biomarkers for cancer treatment major end points, that is overall survival and quality of life, in an appropriate number of patients.

### ACKNOWLEDGEMENTS

We are grateful to all Nurses of Oncologic Radiation Department, San Gerardo Hospital, Monza, for their kind and continuous collaboration and to Linda Cairn, Ph D, for linguistic revision.

### REFERENCES

American Society of Clinical Oncology (1996) Outcomes of cancer treatment for technology assessment and cancer treatment guidelines (Special Article). *J Clin Oncol* **14**: 671–679

Blay JY, Negrier S, Combaret V, Attali S, Goillot E, Merrouche Y, Mercatello A, Ravault A, Tourani JM, Moskovtchenko JF, Philip T and Favrot M (1992) Serum levels of IL-6 as a prognostic factor in metastatic renal cell carcinoma. *Cancer Res* **52**: 3317–3322

Buzio G, De Palma G, Passalacqua R, Potenzoni D, Ferrozzi F, Cattabiani MA, Manenti L and Borghetti A (1997) Effectiveness of very low doses of immunotherapy in advanced renal cell cancer. *Br J Cancer* **76**: 541–544

Chouaib S and Fradelizi D (1982) The mechanism of inhibiton of human IL-2 production. *J Immunol* **129**: 2463–2468

Diamondstone LS, Tollerud DJ, Fuchs D, Wachter H, Brown LM, Maloney E, Kurman C, Nelson DL and Blattner WA (1994) Factors influencing serum neopterin and beta-2 microglobulin levels in a healthy diverse population. *J Clin Immunol* **14**: 368–373

Figlin R, Gitlitz B, Franklin J, Dorey F, Moldawer N, Rausch J, deKernion J and Belldegrun A (1997) Interleukin-2 based immunotherapy for the treatment of metastatic renal cell carcinoma: an analysis of 203 consecutively treated patients. *Cancer J Sci Am* **3**: S92–97

Fischer JR, Schindel M, Stein N, Lahm H, Gallati H, Krammer PH and Drings P (1995) Selective suppression of cytokine secretion in patients with SCLC. *Ann Oncol* **6**: 921–926

Gastl G, Abrams JS, Nanus DM, Oosterkamp R, Silver J, Liu F, Chen M, Albino AP and Bander NH (1993) IL-10 production by human carcinoma cell lines and its relationship to IL-6 expression. *Int J Cancer* **55**: 96–101

Gohring B, Riemann D, Rebmann U, Heynemann H, Schabel J and Langner J (1996) Prognostic value of immunomonitoring of patients with renal cell carcinoma under therapy with IL-2/IFN-α in combination with 5-FU. *Urol Res* **24**: 297–303

Gooding R, Riches P, Dadian G, Moore J and Gore M (1995) Increased soluble interleukin-2 receptor concentration in plasma predicts a decreased cellular response to IL-2. *Br J Cancer* **72**: 452–455

Grimm EA, Mazumder A, Zhang HZ and Rosenberg SA (1982) Lymphokine-activated killer cell phenomenon *J Exp Med* **155**: 1823–1841

Jeal W and Goa KL (1997) Aldesleukin (recombinant Interleukin-2). A review of its pharmacological properties, clinical efficacy and tolerability in patients with renal cell carcinoma. *Bio Drugs* **7**: 285–317

Katsumata N, Eguchi K, Fukuda M, Yamamoto N, Ohe Y, Oshita F, Tamura T, Shinkai T and Saijo N (1996) Serum levels of cytokines in patients with untreated lung cancer. *Clin Cancer Res* **2**: 553–559

Kim US, Papatestas AE and Aufses AH (1976) Prognostic significance of peripheral lymphocyte counts and carcinoembryonic antigens in colorectal carcinoma. *J Surg Oncol* **8** (3): 257–62

Kishimoto T (1989) The biology of interleukin-6. *Blood* **74**: 1–10

Koo AC, Armstrong C, Bochner B, Shimabukuro T, Tso CL, deKernion JB and Belldegrun A (1992) IL-6 and renal cell cancer: production, regulation and growth effects. *Cancer Immunol Immunother* **35**: 97–105

Lissoni P, Viviani S, Santoro A, Barni S and Tancini G (1989) Serum levels of Interleukin-2 in cancer patients: preliminary considerations. *Int J Biol Marker* **4**: 203–206

Lissoni P, Brivio F, Fumagalli L, Galimberti C, Cataldo M, Marsili MT, Brivio O, Barni S, Tancini G and Angelini A (1994) A study of interactions among markers of macrophage functions in metastatic solid tumors: neopterin levels in relation to those of tumor necrosis-alpha and of soluble interleukin-2 receptors. *J Biol Regul Homeost Agents* **8**: 32–35

Lissoni P, Fumagalli L, Rovelli F, Brivio F, Di Felice G and Maiorca F (1998) In vivo stimulation of IL-12 secretion by subcutaneous low-dose IL-2 in metastatic cancer patients. *Br J Cancer* (in press)

Lopez Hanninen E, Kirchner H and Atzpodien J (1996) IL-2 based home therapy of metastatic renal cell carcinoma: risks and benefits in 215 consecutive single institution patients. *J Urology* **155**: 19–25

Maltoni M, Pirovano M, Nanni O, Marinari M, Indelli M, Gramazio A, Terzoli E, Luzzani M, De Marinis F, Caraceni A and Labianca R (1997) Biological indices predictive of survival in 519 italian terminally ill cancer patients. *J Pain Symptom Manage* **13**: 1–9

Monson JRT, Ramsden C and Guillou PJ (1986) Decreased IL-2 production in patients with gastrointestinal cancer. *Br J Surg* **73**: 483–486

Palmer PA, Vinke J, Philip T, Negrier S, Atzpodien J, Kirchner H, Oskam R and Franks CR (1992) Prognostic factors for survival in patients with advanced renal cell carcinoma treated with recombinant IL-2. *Ann Oncology* **3**: 475–480

Palmer PA, Atzpodien J, Philip T, Negrier S, Kirchner H, Von der Maase H, Geertsen P, Evers P, Loriaux E, Oskam R, Roest G, Vinke J and Franks CR (1993) A comparison of 2 modes of administration of rIL-2: continuous intravenous infusion alone versus subcutaneous administration plus IFN-a in patients with advanced renal cell carcinoma. *Cancer Bioth* **8**: 123–135

Riesco A (1970) Five-year cancer cure: relation to total amount of peripheral lymphocytes and neutrophils *Cancer* **25**: 135–140

Rubin LA, Kurman CC and Fritz ME (1985) Soluble IL-2 receptors are released by activated lymphocytes in vitro. *J Immunol* **135**: 3172–3177

Rubin LA, Jay G and Nelson DL (1986) The released interleukin-2 receptor binds interleukin-2 efficiently. *J Immunol* **137**: 3841–3384

Scheid C, Young R and McDermott R (1994) Immune function of patients receiving recombinant human IL-6 in a phase I clinical study: induction of C-reactive protein and IgE and inhibition of natural killer and lymphokine-activated killer cell activity. *Cancer Immunol Immunother* **38**: 119–126

Schoof DD, Douville L, Terashima Y, Richie JP, Batter S and Eberlein TJ (1993) Survival characteristics of metastatic renal cell carcinoma patients treated with lymphokine-activated killer cells plus interleukin-2. *Urology* **41**: 534–539

Scott HR, McMillan DC, Crilly A, McArdle CS and Milroy R (1996) The relationship between weight loss and interleukin-6 in non-small cell lung cancer. *Br J Cancer* **73**: 1560–1562

Sedlmayr P, Rabinowich H, Elder E, Ernstoff MS, Kirkwood JM, Herberman RB and Whiteside T (1991) Depressed ability of patients with melanoma or renal cell carcinoma to generate adherent lymphokine activate killer cells. *J Immunother* **10**: 336–346

© Cancer Research Campaign 1999

Stanley KE (1980) Prognostic factors for survival in patients with inoperable lung cancer. *J Natl Cancer Inst* **65**: 25–32

Tanner J and Tosato G (1991) Impairment of natural killer functions by interleukin-6 increases lymphoblistoid cell tumorigenicity in athymic mice. *J Clin Invest* **88**: 239–247

Tartour E, Dorval T, Mosseri V, Deneux L, Mathiot C, Brailly H, Montero F, Joueux I, Pouillart P and Fridman WH (1994) Serum Interleukin-6 levels and CRP correlate with resistance to IL-2 therapy and poor survival in melanoma patients. *Br J Cancer* **69**: 911–913

Walther MM, Johnson B, Culley D, Shah R, Weber J, Venzon D, Yang JC, Linehan WM and Rosenberg SA (1998) Serum interleukin-6 levels in metastatic renal cell carcinoma before treatment with interleukin-2 correlates with paraneoplastic syndromes but not patient survival. *J Urol* **159**: 718–722

Wojtowicz-Praga S (1997) Reversal of tumor indiced immunosuppression: a new approach to cancer therapy *J Immunother* **20**: 165–177

Yoshino I, Yano T, Murata M, Ishida T, Sugimachi K, Kimura G and Nomoto K (1992) Tumor-reactive T cells accumulate in lung cancer tissues but fail to respond due to tumor cell derived factors. *Cancer Res* **52**: 775–781

Zorn U, Dallmann I, Grosse J, Kirchner H, Poliwoda H and Atzpodien J (1994) Soluble interleukin-2 receptors abrogate IL-2-induced activation of peripheral mononuclear cells. *Cytokine* **6**: 358–364

*© Cancer Research Campaign 1999*
